# Decoding infantile hemangioma: cellular dynamics, molecular signals, and microenvironmental influences

**DOI:** 10.3389/fonc.2025.1675194

**Published:** 2025-09-25

**Authors:** Zongdong Zhu, Xiaoya Jiang, Fang Wang, Lijin Zhao, Qinggao Song

**Affiliations:** ^1^ Oral Disease Research Key Laboratory of Guizhou Tertiary Institution,School of Life Science, Zunyi Medical University, Zunyi, Guizhou, China; ^2^ Hospital of Stomatology, Zunyi Medical University, Zunyi, Guizhou, China; ^3^ Department of General Surgery, Digestive Disease Hospital, Affiliated Hospital of Zunyi Medical University, Zunyi, China

**Keywords:** infantile hemangioma, cell subpopulations, angiogenesis, extracellular matrix, immune microenvironment

## Abstract

Infantile hemangioma (IH), the most prevalent benign vascular tumor in neonates, typically appears several weeks after birth, undergoes rapid proliferation, and subsequently enters a prolonged phase of spontaneous involution. Recent advancements in molecular and cellular biology have revealed increasing evidence that the etiology and progression of IH arise from complex, multi-level interactions involving various factors. In this review, we examine the categorization of IH cells, analyze the pivotal roles of key molecular signaling pathways (e.g., VEGF, HIF, Notch), and elucidate the contributions of immune cells, hypoxia, the extracellular matrix, and exosome-mediated signaling within the tumor microenvironment to the angiogenic processes and regression of IH. These insights will enhance our understanding of IH pathogenesis, thereby laying the groundwork for the development of targeted therapeutic strategies.

## 1 Introduction

**Figure 1:**
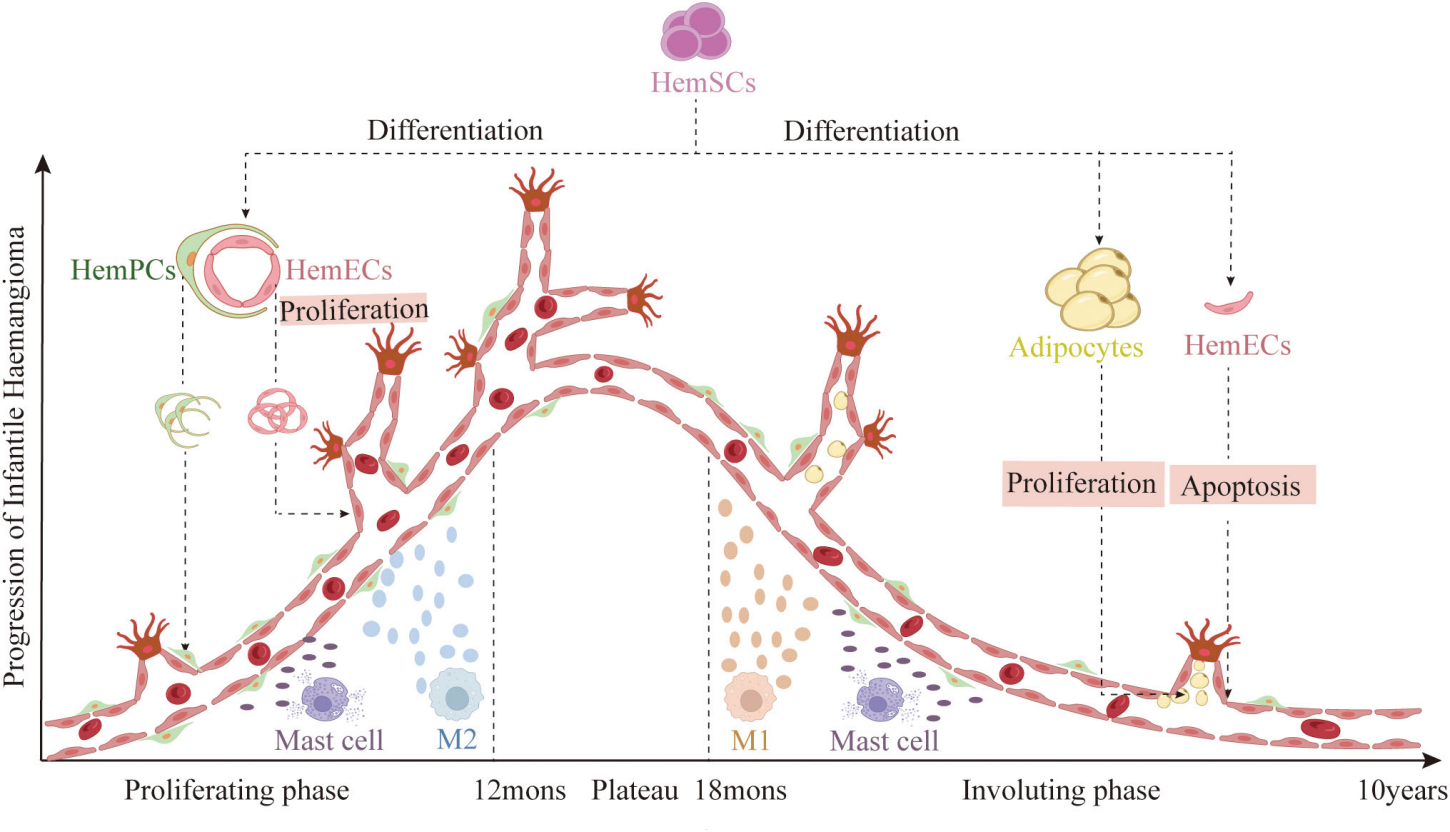
Disease stages of IH: First, a rapid proliferative phase during the first year of life, characterized by abundant endothelial cells forming syncytial masses without a defined vascular architecture. This is followed by a plateau phase of approximately six months, during which growth stabilizes. Next, a gradual involution phase that can last up to ten years, marked by apoptosis of HemECs and differentiation of HemSCs into adipocytes. (Created with BioRender.com).

Infantile hemangioma (IH) is a common benign vascular tumor composed mainly of proliferating endothelial cells. It typically appears as red, sometimes raised, lesions on the skin or subcutaneous tissues. Although IH most often affects the head, neck, and limbs, it can also occur in the viscera or airways (1, 2). IH is not present at birth; it usually emerges within a few weeks after birth, undergoes rapid proliferation during infancy, stabilizes, and then gradually involutes (**Figure 1**). The etiology of IH remains unclear but may involve abnormal fetal blood vessel formation, hypoxia, genetic factors, and environmental influences, with a prevalence of approximately 2%–10% (3–5). IH is more common in premature and low-birth-weight infants, has a female-to-male ratio of about 3:1 (6), and is more prevalent in Caucasians than in Asian or African-American populations (7–9). Although most IH lesions regress naturally, some require aggressive treatment due to ulceration, infection, or impairment of vital organ functions (8). Effective management of IH depends on a thorough understanding of its pathophysiology to develop individualized treatment plans.

IH is typically diagnosed based on its clinical appearance; however, imaging (e.g., ultrasound, MRI) or biopsy may be necessary to exclude other conditions such as lymphangiomas, vascular malformations, or malignant tumors (10–16). Observation is an option for small, non-functional IH, while rapidly growing, ulcerated, or functionally impairing lesions may require early intervention using oral propranolol, topical agents (glucocorticoids or interferon), laser therapy, or surgery (8, 15, 17). Patient-specific, timely decisions are critical to minimize complications and improve outcomes.

A thorough understanding of the cellular, molecular, and microenvironmental mechanisms underlying IH is essential for the development of effective therapeutic strategies. Despite significant advances, the pathogenesis of IH remains incompletely understood. This review integrates current findings to elucidate these interactions from a multidimensional perspective, providing a theoretical foundation for precision treatments, the development of novel therapies, and further research on IH.

## 2 Cell components of IH

**Figure 2:**
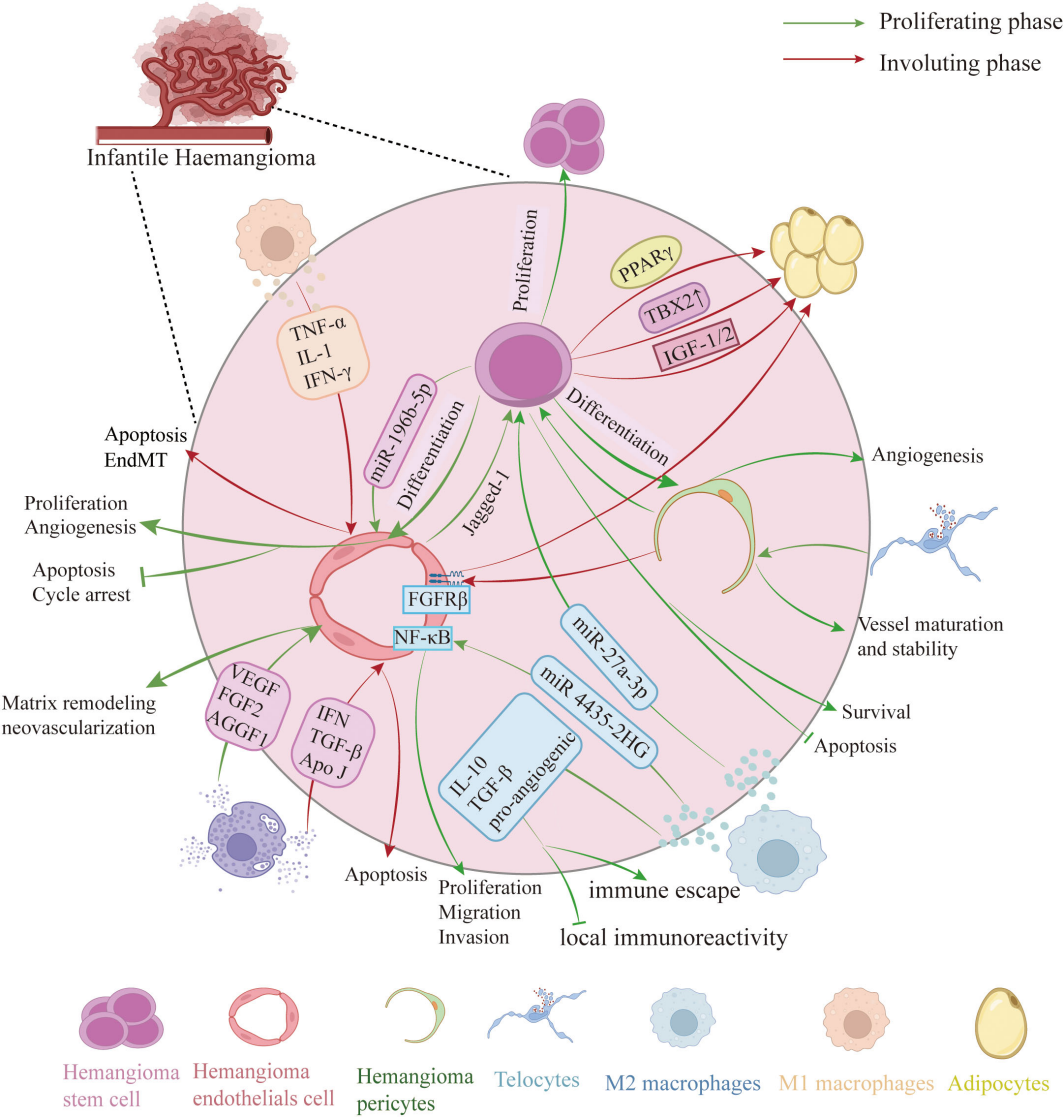
Cellular composition of IH. Proliferative phase: HemSCs differentiate into HemECs, which display enhanced proliferation and migration, reduced apoptosis, and—via Notch–Jagged-1 signaling—further differentiate into HemPCs. M2-polarized macrophages secrete IL-10, TGF-β, pro-angiogenic factors and exosomes that modulate HemSCs and HemECs behavior. Mast cells release VEGF, FGF2, AGGF1 and matrix-degrading enzymes (chymotrypsin, trypsin, MMPs) to remodel the extracellular matrix and drive neovascularization. Involuting phase: HemSCs predominantly become adipocytes. M1 macrophages increase and release TNF-α, IL-1β and IFN-γ to induce HemECs apoptosis or endothelial–mesenchymal transition (EMT), promoting regression. Mast cells produce anti-angiogenic factors (interferon, TGF-β) and apolipoprotein J to trigger early endothelial apoptosis. Telocytes envelop pericytes to support vessel stability and maturation. (Created with BioRender.com).

The development of IH involves dynamic interactions among various cell types (**Figure 2**). Recent research has highlighted the roles of several cellular components in tumorigenesis, angiogenesis, and spontaneous involution (18–20).

### 2.1 Hemangioma stem cells

Hemangioma stem cells (HemSCs) account for approximately 1% of the total cell population (21) and were isolated from IH tissues by Khan et al. Considered the seed cells of IH, they possess multidirectional differentiation potential, enabling them to become hemangioma endothelial cells (HemECs), hemangioma pericytes (HemPCs), or adipocytes in response to microenvironmental cues. This capability underlies rapid neovascularization during the proliferative phase and the formation of residual fibro-adipose tissue during regression (22–25). Isolated from proliferative IH specimens, HemSCs display a mesenchymal morphology *in vitro*, express stem cell markers, e.g., CD133 and CD90, and exhibit high clonogenicity and self-renewal (26, 27). *In vivo*, HemSCs implantation in immunodeficient mice reproduces human IH features, including the formation of Glucose Transporter 1 (GLUT1)-positive vessels and subsequent adipose tissue development (26, 28). As IH regresses, HemSC differentiation toward adipocytes increases, reducing vascular density and generating fibro-fatty tissue—a cellular basis for IH self-limitation (18, 29).

### 2.2 HemECs and HemPCs

HemECs, the most visually apparent component, exhibit typical endothelial morphology, are densely packed, and display irregular arrangements (22). They express markers such as CD31, VE-cadherin, and E-selectin, which aid in diagnosis and indicate similarities to normal endothelial cells. However, HemECs show abnormal proliferation and migration, likely due to low vascular endothelial growth factor 1(VEGFR1) and high VEGFR2 expression (30–33). HemECs can be classified intoGLUT1-positive and GLUT1-negative subtypes. GLUT1-positive HemECs possess stem cell properties and can revert to a mesenchymal phenotype in culture, potentially contributing to IH recurrence (34). In contrast, GLUT1-negative HemECs require supportive cells to establish perfused vessels *in vivo* (35), suggesting that their function depends on both intrinsic gene expression and the surrounding microenvironment.

HemPCs support vascular stability by surrounding endothelial cells (17), and express markers such as Neuron-Glial Antigen 2 (NG-2), Alpha-Smooth Muscle Actin (αSMA), Platelet-Derived Growth Factor Subunit Beta (PDGFRβ), calponin, and Neurogenic locus notch homolog protein 3 (NOTCH3). Notch signaling is upregulated in proliferative IH, promoting HemSC differentiation into pericytes via Notch/Jagged-1 interactions (20, 36, 37). Compared to normal pericytes, HemPCs exhibit enhanced pro-angiogenic properties and reduced contractility, which may contribute to incomplete vessel coverage and increased permeability, thereby facilitating abnormal vessel formation (38). During involution, pericytes mature and stabilize the vasculature while supporting endothelial transformation into adipocytes (17, 39–41).

### 2.3 Additional cellular players

Macrophages in IH are mainly classified into two subtypes. In proliferative IH, M2-polarized macrophages secrete pro-angiogenic factors and exosomes that regulate HemSC and HemEC behavior. During the involution phase, M1-polarized macrophages increase to promote hemangioma regression (42, 43). Mast cells, though fewer during proliferation and more active during early regression, secrete both pro- and anti-angiogenic factors (e.g., VEGF, Fibroblast Growth Factor 2 (FGF2/bFGF), Interferon (IFN), Transforming Growth Factor-Beta (TGF-β)) that modulate angiogenesis and regression, with their activity influenced by the local environment (19, 44). Telocytes (TCs), a distinctive type of interstitial cells, have garnered significant interest because of their distinct morphology and diverse functions. In IH, TCs exhibit overexpression of CD34, PDGFR-α, Vimentin, and Aquaporin-1 (AQP-1), with AQP-1 and PDGFR-α being the most reliable markers for identifying TCs in IH (45). TCs, which closely interact with endothelial cells and pericytes, may regulate intercellular communication and lumen formation (45, 46).

## 3 Molecular mechanisms of IH

**Figure 3:**
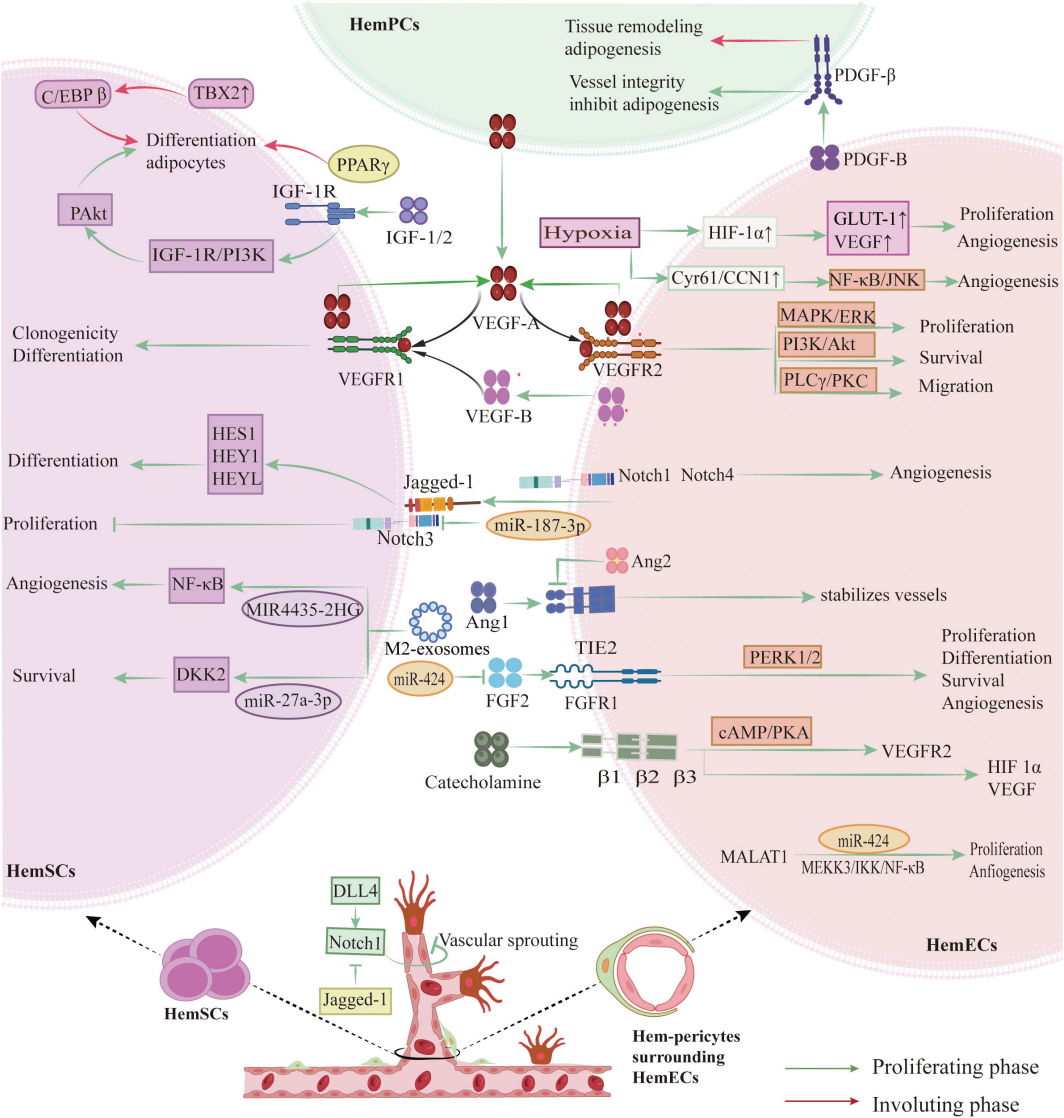
Key signaling pathways and molecular mechanisms in IH pathogenesis. HemECs overexpress VEGFR-2, activating the PI3K/AKT, MEK/ERK, and PLCγ/PKC pathways to drive proliferation, migration, and tube formation. An autocrine VEGFA–VEGFR-2 loop, potentiated by low VEGFR-1 levels, sustains HemECs growth, while paracrine VEGFR-1 signaling directs HemSCs differentiation. Notch signaling—via Notch1/3/4 receptors and Dll4/Jagged1 ligands—controls tip versus stalk cell fate, branching, and pericyte recruitment. β-Adrenergic signaling through β1/2/3 receptors elevates cAMP/PKA and EPAC, upregulating VEGFR-2. The Tie2–Ang1 axis stabilizes vessels via PI3K/EPAC, whereas Ang2 and hypoxia-induced HIF-1α/2α destabilize vessels and enhance VEGFA expression. FGF (bFGF) binding FGFR1 activates ERK1/2 to promote proliferation. Concurrently, IGF-1, IGF-2, PPARγ (including PPARγ2), and TBX2 drive HemSCs adipogenic differentiation, while declining PDGF-B/PDGFR-β signaling permits adipogenesis. (Created with BioRender.com).

The development of IH involves a complex interplay of molecular mechanisms, dysregulated signaling pathways, and cell fate decisions, as summarized in **Table 1**; **Figure 3**.

**Table 1:** Key dysregulated pathways and regulatory axes in infantile hemangioma.

Pathway/regulatory axis	Key components	Dysregulated mechanism	Impact on IH
VEGF/VEGFR	VEGF-A, VEGFR-2, VEGFR-1	Excessive VEGF-A production with constant VEGFR-2 activation in HemECs; VEGFR-1 regulate differentiation of HemSCs to HemECs	Drives rapid endothelial proliferation and abnormal neovascularization.
Notch	Notch receptors (Notch-1, -3, -4), Jagged-1, Dll4	Dynamic shifts in receptor/ligand expression direct the differentiation of HemSCs into mature endothelial cells and pericytes.	Regulates cell differentiation and contributes to complex vessel formation.
β‐Adrenergic	β1, β2, β3 receptors, cAMP,	Activation triggers cAMP-mediated pathways	Enhances angiogenic responses and promotes IH cell proliferation.
SOX18	SOX18, SREBP2, HMGCR	SOX18 regulates endothelial differentiation; aberrant activity sustains mevalonate pathway and angiogenesis; target of R(+)-propranolol	regulator of IH pathogenesis; potential therapeutic target for propranolol and statins
Tie2/Angiopoietin	Tie2, Ang1, Ang2	An imbalance—often marked by upregulated Ang2—leads to competitive inhibition of Tie2, destabilizing vessels by disrupting the Ang1-mediated maintenance of vascular integrity.	Contributes to the formation of immature, leaky vascular networks.
Hypoxia/HIF	HIF-1α	Localized hypoxia stabilizes HIFs, which then drive the transcription of VEGF-A and other proangiogenic factors.	Integrates environmental cues to further potentiate angiogenesis.
FGF2/FGFR1	FGF2, FGFR1	Overexpression of FGF2 leads to enhanced receptor activation and stimulation of downstream proliferative signals.	Promotes endothelial and smooth muscle cell proliferation.
PI3K/AKT/mTOR	PI3K, AKT, mTOR	Aberrant activation reinforces survival and proliferation signals, with upregulation of HIF and VEGF further sustaining the pathway.	Supports sustained cell proliferation and vascular growth.
PDGF-B/PDGFR-β	PDGF-B, PDGFR-β	Active signaling maintains vessel integrity during the proliferative phase; reduced expression during regression facilitates tissue remodeling and adipogenesis.	Regulates pericyte recruitment and vascular wall formation.
IGF	IGF-1, IGF-1R, IGF-2, IGF2R	IGF-1 and IGF-2 activate PI3K/AKT signaling, promoting HemSC adipogenesis; IGF-2 may also inhibit leptin-induced adipogenesis; IGF2R supports proliferation via PCNA, while its loss induces apoptosis in HemECs	Coordinates HemSC differentiation and HemEC survival
PPARγ	PPARγ, PPARγ2, LPL, C/EBPα, COX-2	PPARγ activation downregulates angiogenic factors, suppresses EC proliferation, and promotes HemSC adipogenesis during involution; COX-2 inhibition further enhances adipogenic gene expression	Mediates transition from proliferative vessels to adipose tissue; represents a potential therapeutic target.
TBX2	TBX2, C/EBPβ	TBX2 overexpression enhances C/EBPβ activity, driving HemSC adipocyte differentiation and replacement of vascular tissue with adipose/fibrotic elements	Regulates HemSC fate decisions and may facilitate IH involution.

IH, Infantile Hemangioma; VEGF, Vascular Endothelial Growth Factor; VEGFR, Vascular Endothelial Growth Factor Receptor; HemSCs, Hemangioma Stem Cells; HemECs, Hemangioma endothelial cells; Dll4, Delta-like Ligand 4; cAMP, Cyclic Adenosine Monophosphate; SOX18, SRY-box Transcription Factor 18; SREBP2, Sterol Regulatory Element-Binding Protein 2; HMGCR, 3-Hydroxy-3-Methylglutaryl-Coenzyme A Reductase; Tie2, Tyrosine Kinase with Immunoglobulin-like and EGF-like Domains 2; Ang, Angiopoietin; HIF, Hypoxia-Inducible Factor; FGF2, Fibroblast Growth Factor 2; PI3K, Phosphatidylinositol 3-Kinase; AKT, AKT Serine/Threonine Kinase; mTOR, Mechanistic Target of Rapamycin; PDGF-B, Platelet-Derived Growth Factor Subunit B; PDGFR-β, Platelet-Derived Growth Factor Receptor Beta; IGF, Insulin-Like Growth Factor; PCNA, Proliferating Cell Nuclear Antigen; PPARγ, Peroxisome Proliferator-Activated Receptor Gamma; LPL, Lipoprotein Lipase; C/EBPα, CCAAT/Enhancer-Binding Protein Alpha; TBX2, T-box Transcription Factor 2.

**Table 2:** Surface markers and characteristics of IH cell types.

Cell type	Surface markers and characteristics
HemSCs	Express CD133 and CD90; exhibit high clonogenicity; possess multipotent differentiation capacity (able to differentiate into endothelial cells, pericytes, and adipocytes); drive early tumor proliferation and contribute to fibro-adipose tissue formation during regression.
HemECs	Express CD31, VE-cadherin, and E-selectin; demonstrate abnormal proliferation and migration, contributing to the formation of an aberrant vascular network.
HemPCs	Express NG2, α-SMA, PDGFRβ, Calponin, and NOTCH3; surround endothelial cells to support vascular stability; exhibit strong tubulogenesis but reduced contractility, leading to incomplete vessel coverage and contributing to vascular remodeling.
Immune Cells	Macrophages: M2-type secrete IL-10, TGF-β, and pro-angiogenic factors (supporting immune escape and angiogenesis); M1-type secrete TNF-α, IL-1β, and IFN-γ (promoting apoptosis and remodeling). Mast Cells: Secrete VEGF, FGF2, matrix metalloproteinases, and also release anti-angiogenic factors (IFN, TGF-β).
TCs	Express CD34, PDGFR-α, Vimentin, and Aquaporin-1; involved in intercellular signaling and lumen formation; may modulate the local microenvironment.

HemSCs, Hemangioma Stem Cells Hemangioma Stem Cells; HemECs, Hemangioma Endothelial Cells Hemangioma Endothelial Cells; HemPCs, Hemangioma Pericytes; TCs, Telocytes; M2 Macrophages, Alternatively Activated Macrophages; M1 Macrophages, Classically Activated Macrophages; NG2, Neuron-Glial Antigen 2; α-SMA, Alpha-Smooth Muscle Actin; PDGFR, Platelet-Derived Growth Factor Receptor; NOTCH3, Neurogenic Locus Notch Homolog Protein 3; IL-10, Interleukin-10; TGF-β, Transforming Growth Factor Beta; TNF, Tumor Necrosis Factor; IFN-γ, Interferon Gamma; VEGF, Vascular Endothelial Growth Factor; FGF2, Fibroblast Growth Factor 2.

### 3.1 VEGF/VEGFR pathway

The VEGF/VEGFR pathway is essential for IH (33). In IH, abnormal vascular growth arises from both angiogenesis—the sprouting of new vessels from existing ones—and vasculogenesis, where endothelial progenitor cells differentiate and form primitive vessels; central to both processes is the aberrant activation of the VEGF/VEGFR pathway (47). VEGF-A is highly expressed during the proliferative phase of IH and declines during involution, underscoring its role in sustaining lesion growth (48–50). In proliferative IH, HemECs highly express VEGFR‐2. When VEGF-A binds to VEGFR-2, it activates the Phosphatidylinositol 3-Kinase/Protein Kinase B (PI3K/AKT), Mitogen-Activated Protein Kinase/Extracellular Signal-Regulated Kinase Kinas (MEK/ERK), and Phospholipase C gamma/Protein Kinase C (PLCγ/PKC) cascades, promoting endothelial cell proliferation, migration, and lumen formation, which leads to a dense vascular network (25, 37). HemECs frequently activate autocrine VEGF-A/VEGFR-2 signaling, which stimulates downstream pathway that drive proliferation, migration, and survival (33). Low VEGFR-1 expression in HemECs further amplifies VEGFR-2 signaling, exacerbating abnormal angiogenesis (32, 51). In contrast, HemSCs predominantly express VEGFR-1, which binds VEGF-A to regulate their differentiation into endothelial cells (52). VEGF-B—a VEGFR-1 ligand—is highly expressed in HemECs and similarly promotes HemSC differentiation (53). Together, these paracrine and autocrine VEGF/VEGFR-1 signals synergistically enhance angiogenesis in IH (53). Aberrant VEGF/VEGFR activation not only accelerates endothelial expansion but also stabilizes diseased cells through anti-apoptotic signaling, presenting a clear target for clinical treatment (54–56).

### 3.2 Notch signaling

Notch signaling, which depends on direct cell-to-cell contact, is essential for cell differentiation and vascular maturation; this evolutionarily conserved system governs cell fate decisions and balances proliferation with differentiation (57). In IH, Notch receptors (Notch‐1, Notch‐3, Notch‐4) and ligands (primarily Jagged‐1 and Delta‐like ligand 4 (Dll4)) are abnormally expressed. Upon ligand binding, the Notch intracellular domain is cleaved and translocates to the nucleus to activate transcriptional repressors such as Hairy and Enhancer of Split (HES) and Hairy/Enhancer-of-split related with YRPW motif (HEY), leading to cell cycle exit and maturation (33, 57, 58). In IH tissues, regional variations in Notch expression reflect the differing roles of endothelial and supporting cells. HemSCs are enriched in Notch-3 and downstream targets like HES1, HEY1, and HEYL, priming them for differentiation into both endothelial and mural cells and contributing to the formation of the complex vascular structures typical of IH. In contrast, mature HemECs exhibit higher levels of Notch-1, Notch-4, and Jagged-1 (59). Disruption of Notch receptor–ligand interactions markedly inhibits neovascularization, highlighting its key role in vessel formation and branching (60). Additionally, endothelial-derived Jagged-1 can induce tumor stem cells to acquire a pericyte-like phenotype, which is essential for maintaining vascular wall integrity (37). The balance between Dll4 and Jagged-1 is critical; while Dll4–Notch interactions restrict excessive sprouting by limiting tip cell formation, Jagged-1 promotes vascular branching (61). Experimental evidence shows that blocking Notch signaling leads to significant defects in vessel formation and maturation (60, 62).

### 3.3 β‐adrenergic signaling

The clinical success of propranolol has redirected attention to β‐adrenergic signaling in IH. HemECs express several β‐adrenergic receptor subtypes (β1, β2, and β3), and activation of these receptors by catecholamines triggers the classical Cyclic Adenosine Monophosphate (cAMP) signaling cascade, leading to the activation of Protein Kinase A (PKA) and Exchange Protein Activated by cAMP and an increase in proangiogenic factors such as VEGFR‐2 (63–65). In experimental models, β‐receptor agonists enhance endothelial cell sensitivity to growth factors, while β‐blockers mitigate this effect (66–71). Moreover, β‐adrenergic stimulation may exacerbate angiogenesis in hypoxic conditions by indirectly upregulating Hypoxia‐Inducible Factors-1α (HIF-1α) and VEGF (72, 73). Interestingly, propranolol’s therapeutic effects are not solely due to β‐blockade. Its S(–) enantiomer strongly blocks β‐adrenergic receptors, while the R(+) enantiomer targets the endothelial transcription factor SRY-box transcription factor 18 (SOX18), a master regulator of endothelial differentiation that is aberrantly upregulated in proliferative IH lesions (74–76). Inhibition of SOX18 disrupts transcriptional programs necessary for vessel formation, thereby reducing vascular proliferation. Recent studies indicate that SOX18 also regulates genes in the mevalonate pathway, which is essential for cholesterol biosynthesis and membrane prenylation—key processes for endothelial growth. The R(+) enantiomer selectively inhibits SOX18 independently of β‐adrenergic antagonism, downregulating angiogenic signaling and suggesting potential for drug repurposing (77).

### 3.4 (Tie2)/Angiopoietin signaling

Tie2/Angiopoietin signaling is another key regulator of IH vascular dynamics. The Tie2 receptor on endothelial cells interacts with angiopoietin‐1 (Ang1) and angiopoietin‐2 (Ang2) to control vessel maturation and stability (78, 79). Under normal conditions, Ang1 binding to Tie2 activates pathways such as PI3K/AKT and Mitogen-Activated Protein Kinase (MAPK), promoting endothelial survival, pericyte recruitment, and vessel quiescence (80, 81). In contrast, Ang2—often upregulated during the proliferative phase of IH—acts as a context‐dependent antagonist by competitively inhibiting Tie2 activation, which destabilizes vessels, impairs pericyte recruitment, and leads to the formation of immature, leaky vascular networks (82).

### 3.5 HIFs

HIFs are critical mediators of the cellular response to low oxygen tension and trigger many proangiogenic signals in IH. Rapid hemangioma proliferation often results in localized hypoxia, which stabilizes HIF‐1α and HIF‐2α by preventing their degradation under normoxic conditions (83, 84). Stabilized HIFs translocate to the nucleus, where they induce the transcription of genes such as VEGF‐A and GLUT1 (49, 85, 86) The hypoxia-induced upregulation of VEGF‐A further amplifies autocrine and paracrine signaling loops, driving HemEC proliferation and survival. Additionally, HIFs modulate other key signaling molecules, integrating with pathways such as Notch and Tie2/Angiopoietin to coordinate rapid vascular expansion during the proliferative phase and subsequent vessel maturation and involution as oxygenation improves (87).

### 3.6 bFGF

bFGF is a potent angiogenic factor that stimulates the proliferation of endothelial and smooth muscle cells as well as fibroblast migration (88–90). Its effects are mediated by binding to FGFR1, which triggers receptor autophosphorylation and activates signaling pathways that control cell proliferation, differentiation, survival, and angiogenesis (91, 92). Overexpression of bFGF parallels proliferative hemangioma growth, linking the bFGF/FGFR1 pathway to hemangioma formation, proliferation, and involution (93, 94). Additionally, miR-424 may reduce FGFR1 expression and inhibit the bFGF/FGFR1 pathway, suppressing ERK1/2 phosphorylation and ultimately decreasing cell proliferation, migration, and tube formation (95).

### 3.7 PI3K/AKT/mTOR pathway

The PI3K/AKT/mTOR pathway plays a central role in regulating cell growth, survival, and autophagy (96–98). In IH cells, aberrant activation of this pathway upregulates HIF-1α and VEGF, increasing cellular tolerance to stress (96, 97). Recent studies have shown that SOX4 binds to the promoter of endothelial cell–specific molecule 1 (ESM1), activating the PI3K/AKT pathway and amplifying angiogenic signaling[ (99). Consistently, ex vivo experiments demonstrate that pharmacologic inhibition of this pathway, including the mTOR inhibitor rapamycin, reduces IH cell proliferation and angiogenesis, underscoring its therapeutic potential (100).

### 3.8 Insulin-like growth factor signaling pathway

The insulin-like growth factor (IGF) signaling pathway plays a central role in cell proliferation and insulin sensitivity. IGF-1 binds to its receptor (IGF-1R), a tetramer of two extracellular α-subunits and two transmembrane β-subunits with intrinsic tyrosine kinase activity (101) This interaction activates the PI3K/AKT pathway, increases AKT phosphorylation, and drives HemSCs to differentiate into adipocytes (102). Similarly, IGF-2 promotes HemSCs adipogenesis via the same mechanism. However, one study reported that IGF-2 can inhibit leptin-induced adipogenesis in HemSCs, indicating a context-dependent modulatory role that warrants further investigation (103, 104). In parallel, IGF-2 signaling through IGF2R enhances proliferation by upregulating proliferating cell nuclear antigen (PCNA). Loss of IGF2R, by contrast, weakens PI3K/AKT signaling, reduces PCNA and Bcl-2 expression, and induces apoptosis in HemECs, underscoring IGF2R’s dual role in growth and survival (105).

### 3.9 Peroxisome proliferator-activated receptor γ

Peroxisome proliferator-activated receptor γ (PPARγ) signaling has gained increasing attention in angiogenesis research. Activation of PPARγ exerts anti-angiogenic effects by downregulating angiogenic factors and suppressing endothelial cell migration and proliferation (106, 107). PPARγ agonists, such as thiazolidinediones (TZDs), inhibit angiogenesis by reducing chemotaxis and promoting apoptosis through Erk5 activation (106). In tumor cells, PPARγ ligands also induce growth arrest and apoptosis via the p63 and p73 pathways (108). During involution of IH, PPARγ and its isoform PPARγ2 orchestrate HemSCs differentiation into adipocytes (109). The involuting phase is marked by coordinated upregulation of PPARγ2, lipoprotein lipase (LPL), CCAAT Enhancer-Binding Protein α (C/EBPα), and apolipoprotein A (110). Moreover, cyclooxygenase-2 (COX-2) inhibition may further enhance adipogenic gene expression via the PPARγ/C/EBP axis (111). Collectively, these findings highlight PPARγ as a potential therapeutic target in IH.

### 3.10 T-box transcription factor 2

T-box transcription factor 2 (TBX2), highly expressed in HemSCs, has been proposed as a critical regulator of cell fate decisions (112). TBX2 overexpression augments C/EBPβ activity, promoting adipocyte differentiation of HemSCs, and facilitates the gradual replacement of proliferative vascular tissue with mature adipose and fibrous elements (113).However, current evidence is largely based on *in vitro* studies, with limited *in vivo* validation. It also remains unclear whether TBX2 acts independently or as part of a broader transcriptional network, underscoring gaps in mechanistic understanding.

### 3.11 PDGF-B/PDGFR-β

PDGF family consists of four ligands—PDGF-A, -B, -C, and -D—that bind to the tyrosine kinase receptors PDGFR-α and PDGFR-β (114). The PDGF-B/PDGFR-β system mediates communication between endothelial cells and pericytes, regulating pericyte recruitment and vascular wall formation (115). In proliferative IH, active PDGF signaling maintains vessel integrity by inhibiting adipose differentiation, while reduced PDGFR-β expression during regression facilitates tissue remodeling and adipogenesis (51). Overall, receptor-mediated PDGF signaling appears to constrain involution, but further research is needed to clarify the mechanisms underlying altered PDGF-B expression.

## 4 Role of the microenvironment

### 4.1 Immune and inflammatory mechanisms

Inflammatory cytokines and immune cells play pivotal roles in both the progression and regression of IH. In the proliferative phase, high local concentrations of cytokines such as IL-6, Tumor necrosis factor-α (TNF-α), and IL-1β stimulate the proliferation of HemECs and HemSCs, while enhancing VEGF expression via activation of pathways like Janus Kinase/Signal Transducer and Activator of Transcription (JAK/STAT) and Nuclear factor-kappa B (NF-κB) (116, 117). Allograft inflammatory factor-1(AIF-1) is highly expressed in endothelial cells in most IH samples, which may recruit myeloid cells to the lesion, although the exact source of these cells remains unclear (118). TNF-α exhibits dual effects: it inhibits vascular expansion in regions with insufficient pericytes, whereas in areas with an adequate pericyte population, TNF-α promotes neovascularization, highlighting the modulatory role of pericytes in inflammatory signaling (119).

During the proliferative phase, immune cells in IH are predominantly M2-type macrophages. These cells secrete anti-inflammatory cytokines such as IL-10 and TGF-β, in addition to pro-angiogenic factors, thereby reducing local immunoreactivity and supporting tumor cell immune escape (117, 120). Conversely, in the degenerative phase, the proportion of M1-type macrophages increases; these cells release pro-apoptotic factors such as TNF-α, IL-1β, and IFN-γ, which induce endothelial apoptosis or trigger endothelial–mesenchymal transition (EndMT), thus facilitating tumor regression (117). Moreover, exosomes secreted by M2-type macrophages contain specific non-coding RNAs (e.g., lncRNA mir 4435-2HG) that further enhance HemEC proliferation, migration, and invasion via NF-κB activation (121). Mast cells also exhibit dynamic changes during IH progression. Their numbers are low during the proliferative phase but their enzymatic activity peaks during early regression. Activated mast cells secrete pro-angiogenic factors (e.g., VEGF, FGF2) along with matrix-degrading enzymes (including chymotrypsin, trypsin, and matrix metalloproteinases [MMPs]) to facilitate extracellular matrix (ECM) remodeling and neovascularization (19, 122–126). Additionally, mast cells release anti-angiogenic factors like IFN and TGF-β and produce Apo J, which promotes endothelial apoptosis during the early stages of regression, thereby initiating tumor involution (127).

### 4.2 Hypoxia

A hypoxic microenvironment is a key driver of IH development. During the proliferative phase, rapid tumor cell growth coupled with immature neovascularization results in significant local hypoxia. This low-oxygen state upregulates HIF-1α, which in turn activates downstream genes such as GLUT-1 and VEGF to accelerate HemEC proliferation and neovascularization (49, 85, 86). Furthermore, the protein AIBP, which regulates cholesterol metabolism, promotes cholesterol efflux, destabilizes HIF-1α, reduces VEGF expression, and ultimately inhibits IH growth (128). Cysteine-rich angiogenic inducer 61 (Cyr61/CCN1) is markedly upregulated under hypoxic conditions and localizes primarily to immature microvessels. CCN1 further promotes VEGF-A production through activation of NF-κB and c-Jun N-terminal kinase pathways, creating a positive feedback loop that sustains angiogenesis (129). In addition to promoting angiogenesis, hypoxia relates to metabolic reprogramming in hemangioma cells.

Elevated levels of lncRNA MCM3AP-AS1 enhance glycolysis by increasing glucose uptake and lactate production, thereby providing the energy necessary for rapid cell proliferation. This glycolytic enhancement can be partially reversed by inhibiting HIF-1α, underscoring its role in energy metabolism regulation (130).

### 4.3 ECM

ECM is vital for supporting the vascular endothelium in IH by providing a structural scaffold and regulating angiogenesis. It supports blood vessel formation through adhesive interactions with integrins on endothelial cells, thus maintaining the vascular network (131). Composed of collagen, proteoglycans, and glycoproteins, the ECM is dynamically regulated by its synthesis and degradation, allowing precise control over neovessel formation and maturation. Interactions between the ECM and cells deliver essential signals that govern adhesion, migration, and receptor activation. Alterations in adhesion, increased cell migration, and protease secretion can change vascular permeability, enabling plasma-derived matrix molecules to modify the local ECM composition. Such variations are evident between sites of vessel formation and regions of active angiogenesis. Several studies have shown that changes in the ECM environment correlate with IH progression. For example, differences in ECM composition between the proliferative and involuting phases suggest a causal link between ECM remodeling and angiogenic growth (132). The ECM also influences endothelial responses to angiogenic factors by modulating integrin expression. Adhesion between ECM components and integrins—such as α2β1, α1β1, αvβ3, and α5β1, which bind collagen, fibronectin, and tenascin—is crucial for endothelial tube formation and downstream receptor activation (133–137). In IH, laminin (LN), fibronectin (FN), and vitronectin (VN) are the most frequently studied ECM components (23, 138). For example, LN has been detected in the thickened basement membranes of hemangiomas (139), and the α6-integrin subunit is associated with tumor angiogenesis and cellular invasiveness (140). Despite these insights, further research is needed to clarify other ECM-related factors in IH.

### 4.4 Exosome-mediated signaling

Exosomes, which are extracellular vesicles ranging from 30 to 150 nm in diameter, play crucial roles in intercellular communication within the IH microenvironment. Secreted by HemSCs, HemECs, and immune cells, exosomes carry a variety of bioactive molecules, including miRNAs, lncRNAs, and proteins, which participate in the regulation of IH development.

M2-polarized macrophage-derived exosomes (M2-exos) have been shown to deliver lncRNA MIR4435-2HG to HemECs. This delivery activates the NF-κB signaling pathway via modulation of the HNRNPA1 protein, thereby enhancing cell proliferation, migration, and invasion, and ultimately exacerbating angiogenesis (121). Additionally, M2-exos may transfer miR-27a-3p to HemSCs, leading to the downregulation of Dickkopf-related protein 2 (DKK2). The resulting decrease in DKK2 expression reduces propranolol sensitivity, promotes cell survival, and diminishes apoptosis, offering a potential explanation for treatment resistance in some IH patients (141). Engineered exosomes carrying miR-187-3p have demonstrated the ability to inhibit Notch signaling in HemSCs, resulting in reduced cell proliferation and diminished lumen formation (142). Moreover, Exos derived from IH stem cells are enriched with miR-196b-5p, a molecule that not only promotes HemEC proliferation and angiogenesis but also reduces apoptosis and cell cycle arrest by targeting the CDKN1B gene (143). Targeting exosomal signaling pathways could thus provide a molecular foundation for novel therapies that inhibit pathological angiogenesis and induce tumor regression.

Exosomes act not only as pro-angiogenic mediators but also as potential therapeutic targets. Their molecular cargos may serve as diagnostic or prognostic biomarkers, while Engineered exosomes could be developed as precision drug-delivery systems. Future research should focus on source-specific features, cargo profiles, and translational applications to advance exosome-based therapies for IH.

## 5 Unresolved issues in infantile hemangioma research

### 5.1 Genetic mechanisms

Although familial aggregation and cases in monozygotic twins have been reported (144), most IH cases are sporadic. This suggests that genetic susceptibility, epigenetic regulation, and gene-environment interactions warrant further investigation (145). Early studies suggested a familial basis, with Walter et al. mapping IH to chromosome 5q31–33, which includes genes FGFR4, PDGFRB, and VEGFR-3; variants in these genes were linked to reduced disease risk (146). Somatic mutations in VEGFR2 and VEGFR3 have also been identified in some patients (147), implicating VEGF signaling in IH pathogenesis. However, these findings lack consistent validation in larger cohorts, and their functional significance remains unclear. Single nucleotide polymorphism analyses have suggested a possible association between the VEGF-A rs2010963 G allele and IH susceptibility, but overall evidence remains inconclusive (148).

Increasingly, epigenetic mechanisms—such as DNA methylation, histone modification, and non-coding RNA regulation—are thought to contribute to IH initiation and regression (149). The transcription factor SOX18 and its downstream targets, which regulate vascular development and differentiation, may also influence IH progression via epigenetic pathways and represent promising therapeutic targets (150).

In summary, genetic studies have identified several candidate genes and pathways in IH but no consistent pathogenic mutations. Future investigations should employ multicenter genomic studies, single-cell sequencing, and integrative epigenomic analyses to define the genetic and epigenetic landscape of IH and identify novel susceptibility genes and clinically relevant biomarkers.

### 5.2 Estrogen

IH exhibits a clear female predilection, sparking interest in the role of estrogen and its receptors in its pathogenesis (6). Elevated serum estrogen levels and increased expression of estrogen receptors in IH tissues suggest that estrogen may promote tumor progression through stimulation of endothelial cell proliferation and angiogenesis (6, 19, 151). 17β-estradiol has been shown to bind ER-α in HemSCs, upregulating VEGF-A and enhancing angiogenesis and tumor growth (151). Estrogen also modulates the secretion of multiple angiogenic and anti-angiogenic factors, including FGF-2, IFN-α/β/γ, and TGF-β, which may exert stage-dependent effects during the proliferative and involuting phases of IH (19). Nevertheless, the mechanisms by which estrogen influences IH remain unclear, and its impact on intracellular signaling pathways is debated. Future studies should systematically investigate estrogen’s role at different stages of IH and assess its potential as a therapeutic target.

### 5.3 Safety of propranolol

Propranolol, the primary treatment for IH, has shown substantial efficacy, yet concerns remain regarding its long-term safety, side effects, and potential for recurrence (152). Despite numerous safety studies, there is a lack of multicenter follow-up studies reporting long-term outcomes (153, 154). Some patients experience relapses after treatment cessation, suggesting that propranolol may temporarily impede lesion progression without providing a definitive cure (155, 156). Future large-scale, multicenter randomized controlled trials are needed to assess propranolol’s long-term safety and efficacy. Additionally, exploring combination therapies with other targeted approaches may help reduce recurrence risk.

### 5.4 Mechanism of IH regression

Most hemangiomas gradually degenerate into fibrous adipose tissue after proliferation and stabilization. This regression involves endothelial cell apoptosis, stem cell differentiation into adipocytes, and changes in the local microenvironment (157, 158). However, the precise molecular pathways, regulatory networks, and mechanisms of cell fate determination remain incompletely understood.

### 5.5 Ideal IH model

Current *in vitro* and animal models do not fully mimic the growth and regression of human IH. There is an urgent need to develop comprehensive models that closely reflect human pathology to advance the study of IH mechanisms and therapeutic strategies (159, 160).

### 5.6 Intercellular communication

Intercellular communication in IH involves not only the abnormal behavior of individual cell types but also complex signaling among hemangioma stem cells, endothelial cells, pericytes, and immune cells through cytokines, exosomes, and direct cell-cell interactions (38, 121, 141, 143). The precise mechanisms underlying these interactions remain incompletely elucidated, and further research using techniques such as single-cell sequencing and spatial transcriptomics is needed to fully understand these signaling networks and identify potential molecular targets for therapy.

## 6 Conclusion

Current research indicates that the development of IH is driven by the synergistic effects of multiple cellular (**Table 2**) and molecular factors within a dynamic microenvironment. Abnormally proliferating HemECs and multipotent HemSCs serve as the primary cellular sources of IH formation, while environmental factors such as hypoxia, inflammation, ECM, and exosome-mediated intercellular communication critically regulate tumor growth, angiogenesis, and eventual regression (3, 17, 18, 84, 161).

With growing insight into IH biology, several molecules have emerged as promising therapeutic targets. SOX18, a key transcription factor in vascular development and endothelial differentiation, has been implicated in abnormal endothelial proliferation in IH. Pharmacologic inhibition of SOX18, including the non–β-adrenergic effects of R(+)-propranolol and repurposing of statins via the SOX18–mevalonate pathway axis, highlights its translational potential (74, 77, 150). Exosomes, as central mediators of intercellular communication and angiogenesis, represent potential intervention points through modulation of their release or function. HIF-1α, a major driver of VEGF expression in hypoxic niches, may provide an opportunity for early control of IH proliferation (87). In addition, hormonal signaling and immune microenvironmental regulation may enable more individualized treatment strategies.

In conclusion, IH is a complex vascular anomaly regulated by a dynamic network of cellular and molecular mechanisms. Continued exploration of its genetic, metabolic, and immunologic drivers will provide a foundation for future innovation in vascular biology and therapy. Progress will require multidisciplinary approaches combining advanced molecular technologies with rigorous clinical research. Such efforts will be critical to resolving existing controversies, validating emerging therapeutic targets, and accelerating the translation of mechanistic discoveries into precision treatments for IH.
